# Multivariate Multiscale Complexity Analysis of Self-Reproducing Chaotic Systems

**DOI:** 10.3390/e20080556

**Published:** 2018-07-27

**Authors:** Shaobo He, Chunbiao Li, Kehui Sun, Sajad Jafari

**Affiliations:** 1 School of Physics and Electronics, Central South University, Changsha 410083, China; 2 Jiangsu Collaborative Innovation Center of Atmospheric Environment and Equipment Technology (CICAEET), Nanjing University of Information Science & Technology, Nanjing 210044, China; 3 Jiangsu Key Laboratory of Meteorological Observation and Information Processing, Nanjing University of Information Science & Technology, Nanjing 210044, China; 4 Department of Biomedical Engineering, Amirkabir University of Technology, 424 Hafez Ave., Tehran 15875-4413, Iran

**Keywords:** multiscale multivariate entropy, multistability, self-reproducing system, chaos

## Abstract

Designing a chaotic system with infinitely many attractors is a hot topic. In this paper, multiscale multivariate permutation entropy (MMPE) and multiscale multivariate Lempel–Ziv complexity (MMLZC) are employed to analyze the complexity of those self-reproducing chaotic systems with one-directional and two-directional infinitely many chaotic attractors. The analysis results show that complexity of this class of chaotic systems is determined by the initial conditions. Meanwhile, the values of MMPE are independent of the scale factor, which is different from the algorithm of MMLZC. The analysis proposed here is helpful as a reference for the application of the self-reproducing systems.

## 1. Introduction

Since the behaviors of dynamic systems with coexisting attractors depend on the initial conditions, multistable systems have been extensively studied [1,2,3,4,5]. Multistability in circuit implementation [6], synchronization [7], image encryption [8] and neural networks [9] have also aroused much interest. Multistable systems can have a limited number of coexisting attractors [10] or even infinitely many attractors [11,12]. Specifically, Li et al. proposed a class of self-reproducing systems (one case of conditional symmetry) giving one-dimensional infinitely many attractors [11] and a unique case with a two-dimensional lattice of infinitely many strange attractors by introducing periodic trigonometric functions into a two-dimensional offset-boostablesystem [12].

However, the dynamics of these systems are mainly analyzed by means of a bifurcation diagram, Lyapunov exponents (LEs) and the phase trajectory analysis. Generally, higher complexity means that the time series is closer to noise and consequently leads to better security for real applications. Complexity measuring methods can be used to reflect the dynamics and complexity of time series in chaotic systems and provide an effective means for parameter selection of chaotic systems in real applications. From this point of view, much research on the complexity of chaotic systems has been carried out [13,14,15,16,17,18]. For example, Balasubramanian et al. [15] classified periodic, chaotic and random sequences based on approximate entropy and Lempel–Ziv complexity measures. He et al. [17,18] analyzed the complexity of multi-scroll chaotic systems and fractional-order chaotic systems. Complexity measuring of chaotic systems is an important issue in the nonlinear research community, among which designing multivariate complexity measures for a chaotic attractor is a hot topic.

In fact, phase space analysis is one of the most useful methods for the explanation of the long term dynamics of multivariate systems [19]. Phase-space reconstruction can reflect the asymptotic nature of the interconnected time series, which are responsible for the original dynamics. Most of the current multivariate complexity algorithms are designed based on this, such as multivariate sample entropy (MvSampEn) [20] and multivariate neighborhood sample entropy (MN-SampEn) [21]. However, it is difficult to choose the embedding dimension and the delay parameter. An alternative way to measure the complexity of the system is to resort to its phase space directly. Recently, He et al. [22] proposed multivariate permutation entropy and applied it to the complexity analysis of chaotic attractors. Meanwhile, since multiscale coarse-graining [23] on time series could lead to better complexity measuring results, multiscale complexity algorithms are applied to analyze the complexity of nonlinear time series [18,23,24,25]. In this paper, combining the process of multiscale coarse-graining, multivariate multiscale complexity measure algorithms are designed to analyze the complexity for self-reproducing chaotic systems. It is necessary to analyze the complexity of attractors of this class of chaotic systems under different initial conditions for its multistability. Multiscale multivariate complexity measuring algorithms are designed to achieve this goal. The rest of this article is organized as follows: In Section 2, MMPE and MMLZC are designed. In Section 3, the complexity of different kinds of self-reproducing chaotic systems is analyzed. Finally, concluding remarks is presented in Section 4.

## 2. Designing the Complexity Measuring Algorithms

Compared with other nonlinear time series analysis methods, complexity measuring methods just need a segment of time series or multiple time series from the system and are robust to the algorithm parameters. Meanwhile, it is more convenient to analyze the characteristics of time series by employing complexity measuring algorithms. In this section, the complexity of three-dimensional (3D) chaotic systems is analyzed with the main purpose to measure the complexity of the attractors. Thus, it is necessary to design multivariate complexity measuring algorithms.

### 2.1. Data Processing and Quantification

Step 1: Normalization of time series. For given time series $\left\{x_{j}\left(n\right),n=1,2,3,\cdots ,N,j=1,2,\cdots ,d\right\}$, where *d* is the number of time series or the dimension of the chaotic system. Since amplitudes of different time series are different, normalization processing is necessary. The normalization function is given by:
  (1) $${\tilde{x}}_{j}\left(n\right)=\frac{x_{j}\left(n\right)-\min\left(x_{j}\right)}{\max\left(x_{j}\right)-\min\left(x_{j}\right)}.$$
Step 2: Coarse graining. To design multiscale complexity measuring algorithms, the multiscale coarse-grained processing should be carried out firstly. For the *j*-th time series, its consecutive coarse-grained time series is constructed by [23]:
  (2) $$y_{j}^{\tau }\left(k\right)=\frac{1}{s}\sum_{i=(k-1)s+1}^{ks}{\tilde{x}}_{j}\left(i\right),$$
  where 1≤k≤⌊N/τ⌋ and τ is the scale factor that represents the length of the non-overlapping windows.
Step 3: Data quantification. For the given *k* and scale factor τ, [$y_{1}^{\tau }\left(k\right)$, $y_{2}^{\tau }\left(k\right)$, ⋯, $y_{d}^{\tau }\left(k\right)$] can be modeled as a pattern by introducing the idea of the Bandt–Pompe pattern [26]. Obviously, there are d! possible patterns. Let the pattern space be given by $\Lambda =\left\{\pi_{1},\pi_{2},\cdots ,\pi_{d!}\right\}$, and thus, a pattern series $\left\{\Psi^{\tau }\left(k\right):\Psi^{\tau }\left(k\right)\in \Lambda ,k=1,2,\cdots ,\left\lfloor N/\tau \right\rfloor \right\}$ can be obtained. Moreover, let $\pi_{l}=l\left(l=1,2,\cdots ,d!\right)$; we can get a quantification pattern series, which is given by $\left\{\Phi^{\tau }\left(k\right):\Phi^{\tau }\left(k\right)\in N,k=1,2,\cdots ,\left\lfloor N/\tau \right\rfloor \right\}$.

To understand the above process better, here we illustrate how $\pi_{s}\left(s=1,2,\cdots ,d!\right)$ are obtained firstly. Take a 3D chaotic system as an example, the parameter d=3. Thus, there are six possible order patterns under the *k*-th point, and they are shown as below:

(3) $$\left\{\begin{array}{c} \left\{\pi_{1}:y_{1}^{\tau }\left(k\right)\leq y_{2}^{\tau }\left(k\right)\leq y_{3}^{\tau }\left(k\right)\right\}\\ \left\{\pi_{2}:y_{1}^{\tau }\left(k\right)\leq y_{3}^{\tau }\left(k\right)\leq y_{2}^{\tau }\left(k\right)\right\}\\ \left\{\pi_{3}:y_{2}^{\tau }\left(k\right)\leq y_{1}^{\tau }\left(k\right)\leq y_{3}^{\tau }\left(k\right)\right\}\\ \left\{\pi_{4}:y_{2}^{\tau }\left(k\right)\leq y_{3}^{\tau }\left(k\right)\leq y_{1}^{\tau }\left(k\right)\right\}\\ \left\{\pi_{5}:y_{3}^{\tau }\left(k\right)\leq y_{1}^{\tau }\left(k\right)\leq y_{2}^{\tau }\left(k\right)\right\}\\ \left\{\pi_{6}:y_{3}^{\tau }\left(k\right)\leq y_{2}^{\tau }\left(k\right)\leq y_{1}^{\tau }\left(k\right)\right\} \end{array}\right.,$$

For example, let $\left[y_{1}^{\tau },y_{2}^{\tau },y_{3}^{\tau }\right]$ = $\left[1,3,4\right]$; it can be defined as order pattern $\pi_{1}$; while if $\left[y_{1}^{\tau },y_{2}^{\tau },y_{3}^{\tau }\right]$ = $\left[1,4,3\right]$, it belongs to another kind of pattern, which can be classified as $\pi_{2}$. Secondly, suppose that we have three short time series, namely $\left\{y_{1}^{\tau }=1,2,3,4,5\right\}$, $\left\{y_{2}^{\tau }=3,3,1,5,4\right\}$ and $\left\{y_{3}^{\tau }=5,1,4,3,2\right\}$; the conducted pattern series $\left\{\Psi^{\tau }\left(k\right)=\pi_{1},\pi_{5},\pi_{3},\pi_{5},\pi_{6}\right\}$, and the quantification pattern series is $\left\{\Phi^{\tau }\left(k\right)=1,5,3,5,6\right\}$.

### 2.2. Complexity Measuring Algorithms

Permutation entropy [26] can be calculated based on the comparison of neighboring values (quantification pattern series) by combing with the concept of Shannon entropy. It is particularly useful in the presence of dynamical or observational noise. However, Lempel–Ziv complexity (LZC) [27,28] is not based on the probabilistic of the symbols, but on the way that these symbols are repeated along the sequences. Based on the quantification pattern series, multiscale multivariate permutation entropy and multiscale multivariate Lempel–Ziv complexity can be designed.

#### 2.2.1. Multiscale Multivariate Permutation Entropy

The probability distribution $P^{\tau }=p^{\tau }\left(\pi_{l}\right)|l=1,2,\cdots ,d!$ associated with the quantification pattern series Φτk:Φτk∈N,k=1,2,⋯,floorNNττ is defined by:

(4) pτ(πl)=#{k|k≤floorNNττ,Φτk=l}floorNNττ,

where the symbol # stands for “number” and l=1,2,3,⋯,d!. According to the definition of Shannon entropy, MMPE is defined as:

(5) $$MM\mathrm{PE}\left(x,\tau \right)=-\frac{1}{\ln(d!)}\sum_{s=1}^{d!}p^{\tau }\left(\pi_{s}\right)\ln\left(p^{\tau }\left(\pi_{s}\right)\right),$$

Obviously, larger MMPE values mean the time series is more complex.

#### 2.2.2. Multiscale Multivariate Lempel–Ziv Complexity

First of all, Lempel–Ziv complexity [28] is described, and the steps are shown as follows.

Step 1: Suppose that the quantification pattern series is $\left\{\Phi^{\tau }\left(k\right)=s_{1},s_{2},s_{3},\cdots ,s_{N}\right\}$. Let *S* and *Q* be two character strings.
Step 2: For the step *n* (n=1,2,3,⋯,N), let $S=\left(s_{1},s_{2},s_{3},\cdots ,s_{n}\right)$, and $Q=s_{n+1}$ or $Q=\left(s_{n+1},s_{n+2},\cdots ,s_{n+k}\right)$, then we get:
  (6) $$SQ=\left(s_{1},s_{2},s_{3},\cdots ,s_{n},s_{n+1}\right),$$
  or:
  (7) $$SQ=\left(s_{1},s_{2},s_{3},\cdots ,s_{n},s_{n+1},s_{n+2},\cdots ,s_{n+k}\right).$$
  Define:
  (8) $$SQ_{v}=\left(s_{1},s_{2},s_{3},\cdots ,s_{n}\right).$$
  or:
  (9) $$SQ_{v}=\left(s_{1},s_{2},s_{3},\cdots ,s_{n},s_{n+1},s_{n+2},\cdots ,s_{n+k-1}\right).$$
  If there exist an i(1≤i≤n) and the following relationship is satisfied:
  (10) $$\left(s_{n+1},s_{n+2},\cdots ,s_{n+k}\right)=\left(s_{i},s_{i+1},\cdots ,s_{i+k-1}\right).$$
  it means that *Q* is a duplicate of $SQ_{v}$. Then, the size of *Q* should increase by one, and the above operation is carried out again until $Q\notin SQ_{v}$. When *Q* does not belong to $SQ_{v}$, we call *Q* an “insertion”. When an “insertion” is found, we place a “·” behind *S*. Repeat the above operations until n=N.
Step 3: In Step 2, we obtained a series of dots; thus, we can calculate the number of dots and denote the complexity as c(n).
Step 4: According to [27], Lempel–Ziv complexity will reach a stable value, which is given by:
  (11) $$LZ_{Stable}=\lim_{n\to \infty }c\left(n\right)=\frac{n}{\log_{2}\left(n\right)}.$$
  where $LZ_{Stable}$ is the stable complexity measure value of a finite long time series. Thus, the normalized multiscale multivariate Lempel–Ziv complexity is defined as:
  (12) $$MMLZC=\frac{c\left(n\right)}{LZ_{stable}},\left(0\leq MMLZC\leq 1\right).$$

The scale factor is given by the quantized time series $\left\{\Phi^{\tau }\left(k\right)=s_{1},s_{2},s_{3},\cdots \right\}$.

Here, an example is given to show the steps of the LZC algorithm. Suppose that the quantized time series with length n=7 is $\left\{\Phi^{\tau }=1,2,3,2,3,2,3\right\}$. Firstly, $\Phi^{\tau }$ is converted to a string time series.

n=1, S=1, Q=2, $SQ_{v}=1$. Because $Q\notin SQ_{v}$, then *Q* is an insertion S=1·2.

n=2, S=12, Q=3, $SQ_{v}=12$. Because $Q\notin SQ_{v}$, then *Q* is an insertion S=1·2·3.

n=3, S=123, Q=2, $SQ_{v}=123$. Because $Q\in SQ_{v}$, then *Q* is a copy of S=1·2·32.

n=4, S=1232, Q=23, $SQ_{v}=123$. Because $Q\in SQ_{v}$, then *Q* is a copy of S=1·2·323.

Continue the above steps until n=7; we can get S=1·2· 32323. This means that the time series can be divided into three parts; thus, $c\left(n\right)=3$. According to Equation (12), LZC=0.8159. However, this measuring result is not satisfying since the length of this example is not suitable. To make the measuring results stable, the length of the sequence should be larger than 3600 [29].

#### 2.2.3. Process for Complexity Measuring

Here, take a 3D chaotic system as an example, the process of the complexity measure is illustrated as follows. The steps to analyze the complexity of attractors and the representation of the result in the complexity vs. entropy map are described.

**Figure 1:**
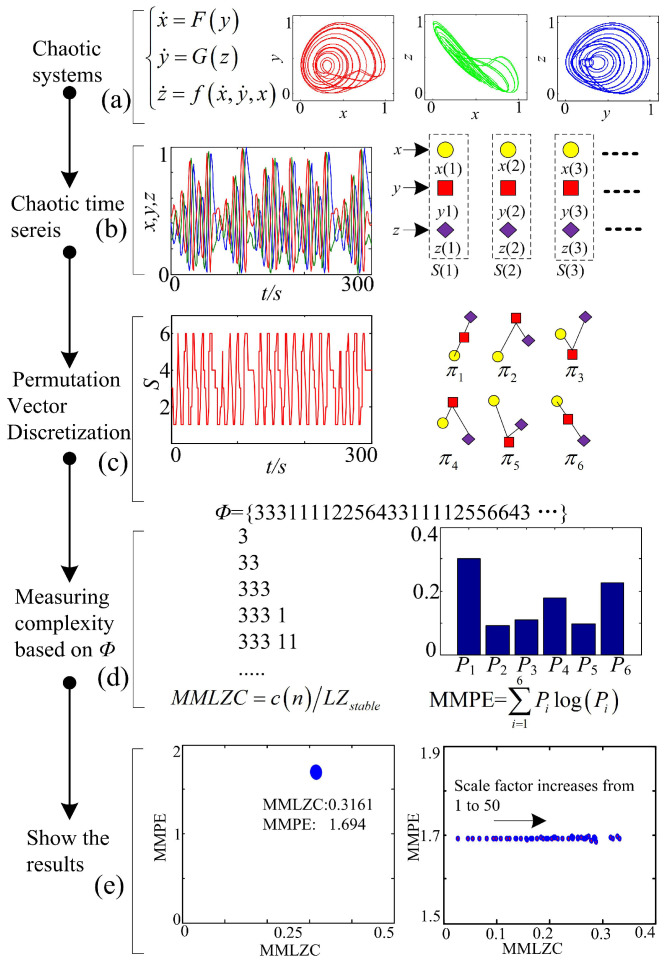
Steps to analyze the complexity of a chaotic system through the multiscale multivariate Lempel–Ziv complexity (MMLZC) and multiscale multivariate permutation entropy (MMPE) and algorithms. (**a**) Chaotic systems; (**b**) Chaotic time series; (**c**) Permutation Vector Discretization; (**d**) Measuring complexity based on Φ; (**e**) Show the results.

Step 1: Figure 1a. Solve the chaotic system and observe the state of the system based on the phase diagrams, preliminarily.
Step 2: Figure 1b. Cut three segments of chaotic time series, which are the three state variables of the 3D chaotic system. Data processing and coarse graining are carried out by employing the method given in the Section 2.1.
Step 3: Figure 1c. Quantize the scaled time series using the Bandt–Pompe approach; thus, a symbol time series is obtained.
Step 4: Figure 1d. Estimate the MMLZC and MMPE according to the obtained sequence, where the steps of MMPE and MMLZC are shown in Section 2.2.1 and Section 2.2.2, respectively.
Step 5: Figure 1e. Illustrate the complexity measuring results with different figures. Here, the two measures are shown in the MMPLC-MMPE plane.
  Note that we also illustrate the complexity with MMLZC and MMPE as shown by the curve and surfaces for comparison.

## 3. Complexity Analysis of Self-Reproducing Chaotic Systems

Multistable systems have multiple solutions under different initial conditions, and the self-reproducing system is a new kind of multistable system with infinitely many coexisting attractors by reproducing themselves along particular dimensions or directions. It should be pointed out that all of those coexisting attractors in a system share the same Lyapunov exponents, and the infinitely many attractors in self-reproducing chaotic systems are triggered by the initial condition [11,12]. Therefore, it is interesting to check whether those coexisting attractors have the same complexity. Here, the complexity of a one-directional self-reproducing system and two-directional self-reproducing system is analyzed by means of MMPE and MMLZC.

### 3.1. Case A: One-Directional Self-Reproducing System

**Figure 2:**
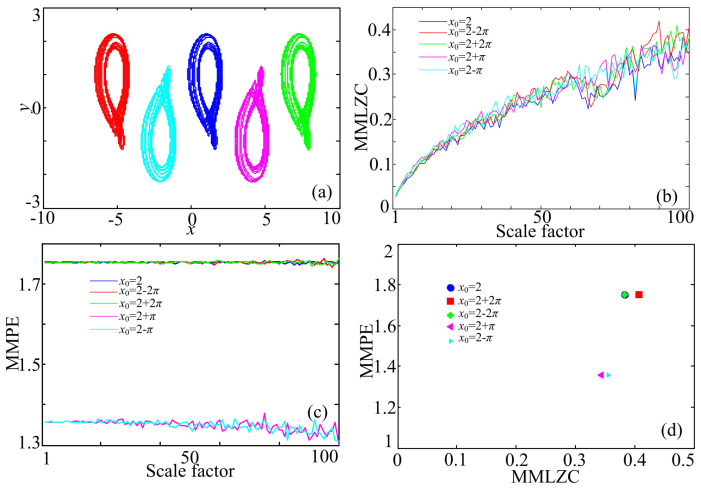
Coexisting attractors and complexity analysis results of System (13). (**a**) Coexisting attractors; (**b**) MMLZC; (**c**) MMPE; (**d**) MMLZC-MMPE plot.

For the self-reproducing system [10],

(13) $$\left\{\begin{array}{c} \dot{x}=\left|y\right|-1\\ \dot{y}=z\\ \dot{z}=F\left(x\right)-by-az \end{array}\right.,$$

where *x*, *y* and *z* are the state variables, *a* and *b* are the bifurcation parameters and $F\left(x\right)=A\cos\left(x\right)$, A=1.55. Fix a=0.6, b=1. Set the initial conditions $\left(x_{0},y_{0},z_{0}\right)=\left(2-2\pi ,0,-1\right)$, (2−π,0,−1), (2,0,−1), (2+π,0,−1) and (2+2π,0,−1); five attractors under different initial conditions are shown in Figure 2a. The red attractor is plotted with $\left(x_{0},y_{0},z_{0}\right)=\left(2-2\pi ,0,-1\right)$; the cyan attractor is plotted with $\left(x_{0},y_{0},z_{0}\right)=\left(2-\pi ,0,-1\right)$; the blue attractor is plotted with $\left(x_{0},y_{0},z_{0}\right)=\left(2,0,-1\right)$; the mauve attractor is plotted with $\left(x_{0},y_{0},z_{0}\right)=\left(2+\pi ,0,-1\right)$; and the green attractor is plotted with $\left(x_{0},y_{0},z_{0}\right)=\left(2+2\pi ,0,-1\right)$. The MMLZC and MMPE analysis results are shown in Figure 2b,c, respectively. It is shown in Figure 2b that multivariate LZC increases with the scale factor τ. However, different complexities overlap with each other. This indicates that the Lempel–Ziv complexity of different attractors is of the same level. However, Figure 2c shows that the complexity of multivariate PE does not increase with the scale factor. This means that the complexity of an attractor does not increase with the scale factor in the sense of multivariate PE. Moreover, as shown in Figure 2c, the complexity of the attractors with $\left(x_{0},y_{0},z_{0}\right)=\left(2-\pi ,0,-1\right)$ and (2+π,0,−1) is lower than that of other cases. Here, let the scale factor τ for MMLZC be 100 and for MMPE be one. The MMLZC-MMPE plot in Figure 2d shows that the complexity of attractors with $\left(x_{0},y_{0},z_{0}\right)=\left(2-\pi ,0,-1\right)$ and (2+π,0,−1) is relatively lower than the other cases.

**Figure 3:**
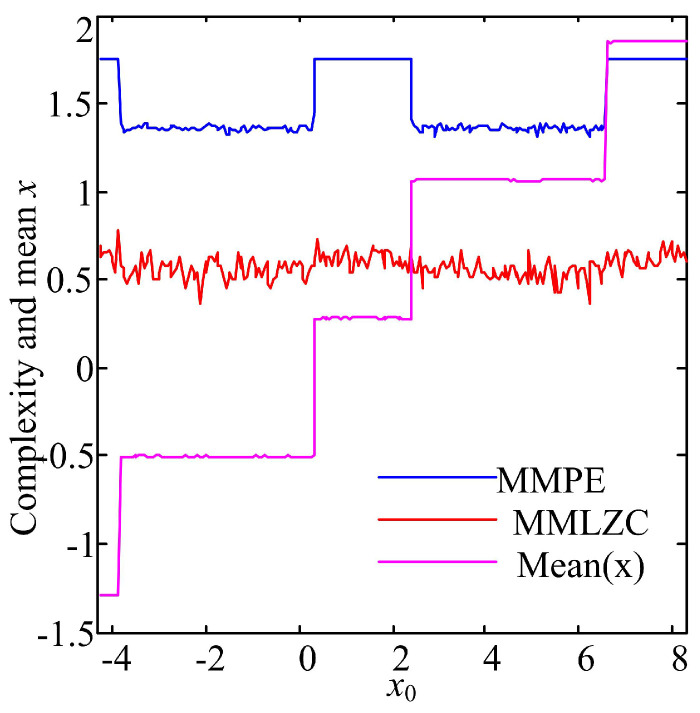
Complexity analysis result and mean value of *x* of System (13).

Fix $y_{0}=0$ and $z_{0}=-1$, and vary $x_{0}$ from 2−2π to 2+2π with a step size of 0.001. The complexity results are shown in Figure 3; MMPE of the chaotic system under different initial conditions varies with the values of $x_{0}$. Lines of MMPE and *x* smoothly evolve according to $x_{0}$. MMPE has the same variation tendency with the mean value of state variable *x*. Different from MMPE, MMLZC shows some robustness with the initial condition. As a matter of fact, it is indicated in [10] that different attractors have the same LEs (0.00285, 0, −0.6285). However, it should be noted that the complexity of the system still depends on the initial condition.

**Figure 4:**
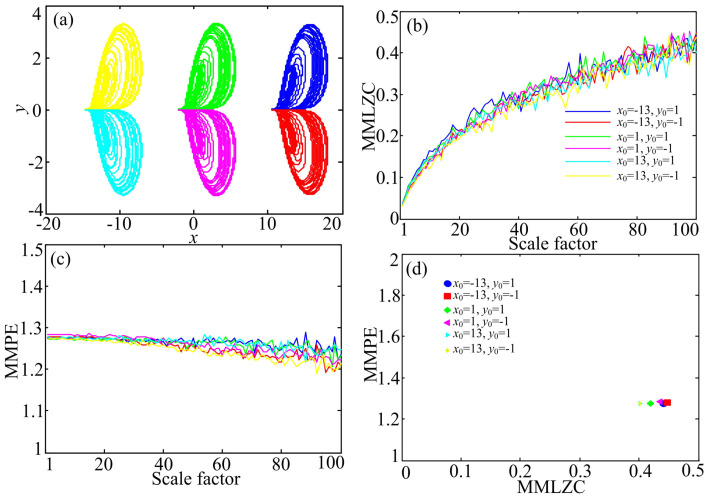
Coexisting attractors and complexity analysis results of System (14). (**a**) Coexisting attractors; (**b**) MMLZC; (**c**) MMPE; (**d**) MMLZC-MMPE plot.

Another one-directional self-reproducing chaotic system was defined by [10]:

(14) $$\left\{\begin{array}{c} \dot{x}=az+y^{2}-1\\ \dot{y}=byz\\ \dot{z}=-A\sin\left(Bx\right)-z \end{array}\right.,$$

where a=2.8, b=4, A=2.2 and B=0.5. When the initial conditions are given by $\left(x_{0},y_{0},z_{0}\right)=\left(-13,1,0\right),\left(-13,-1,0\right),\left(1,1,0\right),\left(-1,1,0\right),\left(13,-1,0\right)$ and (−13,1,0), coexisting attractors are shown in Figure 4a. The position of chaotic attractors is decided by the values of $x_{0}$ and $y_{0}$. When $\left(x_{0},y_{0},z_{0}\right)=\left(-13,1,0\right)$ and (−13,−1,0), the yellow and the cyan attractors are plotted in the left part of Figure 4a. When $\left(x_{0},y_{0},z_{0}\right)=\left(1,1,0\right)$ and (−1,1,0), the green and the mauve attractors are shown in the middle of Figure 4a, while when $\left(x_{0},y_{0},z_{0}\right)=\left(13,-1,0\right)$ and (−13,1,0), the red and the blue attractors are illustrated in the right part. The MMPE and MMLZC complexities of these attractors are calculated as shown in Figure 4b–d. According to Figure 4, we see that the multivariate LZC increases with the scale factor, but MMPE does not increase with the variation of the scale factor. According to the above analysis, the complexity of different attractors is at about the same level.

**Figure 5:**
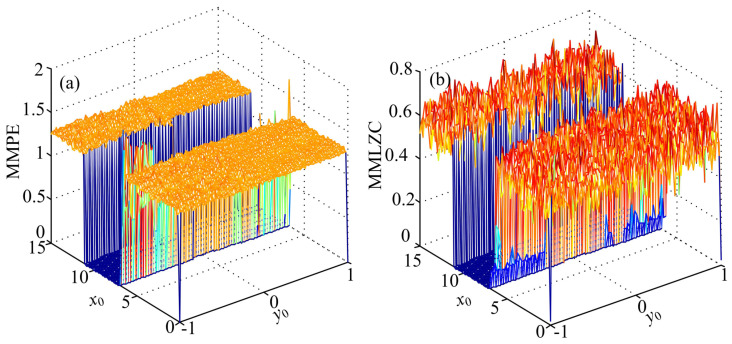
Complexity analysis results of System (14) with simultaneous variations of $x_{0}$ and $y_{0}$. (**a**) MMPE analysis result; (**b**) MMLZC analysis result.

Let $x_{0}$ vary from 0 to 15 with a step size of 0.15 and $y_{0}$ vary from −1 to 1 with a step size of 0.02. MMLZC and MMPE of System (13) are computed, and the complexity of System (13) with simultaneous variations of $x_{0}$ and $y_{0}$ is analyzed. It is shown in Figure 5 that the complexity of System (14) does not change when $y_{0}$ takes different values and $x_{0}$ varies in the interval $x_{0}\in \left[6.8183,11.2122\right]$. As a matter of fact, when $x_{0}\in \left[6.8183,11.2122\right]$, the solution of the system goes to infinity, which means that the system is divergent.

### 3.2. Case B: Two-Directional Self-Reproducing System

**Figure 6:**
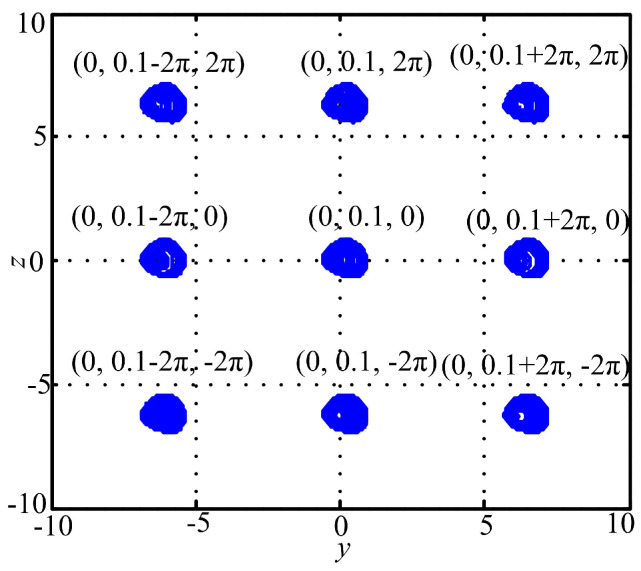
Coexisting attractors of System (15) with different initial conditions.

Li et al. [12] designed a two-directional self-reproducing chaotic system,

(15) $$\left\{\begin{array}{c} \dot{x}=\sin\left(y\right)\\ \dot{y}=a\sin\left(z\right)\\ \dot{z}=-\sin\left(y\right)-b\sin\left(z\right)-x+x^{2} \end{array}\right.,$$

where *x*, *y* and *z* are the state variables and a and bare the system parameters. When a=1.05 and b=0.5, the system gives chaos with LEs (0.0890,0,−0.5808). Figure 6 illustrates the coexisting attractors of System (15) with initial conditions given by $\left(x_{0},y_{0},z_{0}\right)=\left(0,0.1-2k\pi ,2l\pi \;\left(k,l=-1,0,1\right)\right)$. It shows that attractors are distributed in a grid distribution according to the given initial conditions.

**Figure 7:**
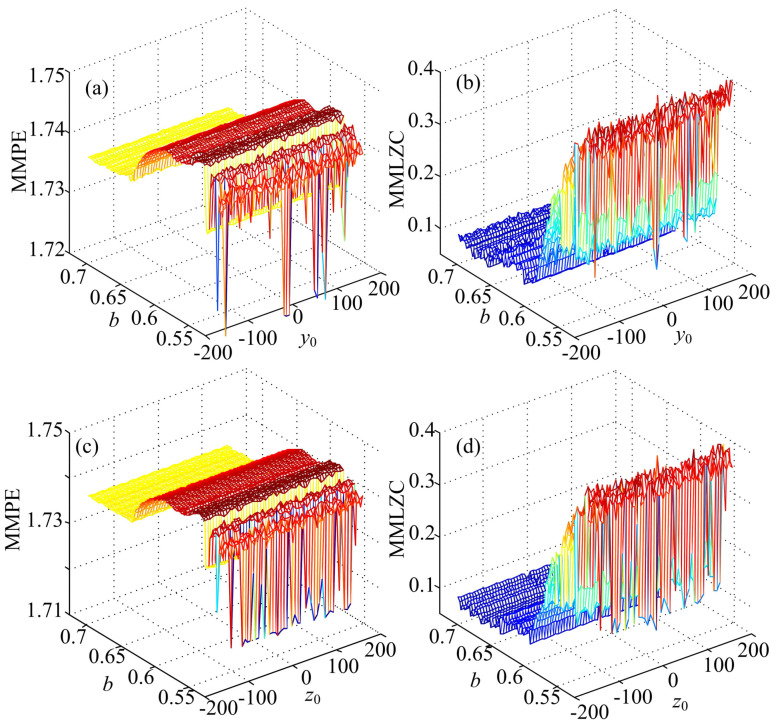
Complexity analysis results of System (15). (**a**) MMPE with $y_{0}$ and *b*; (**b**) MMPE with $y_{0}$ and *b*; (**c**) MMPE with $y_{0}$ and *b*; (**d**) MMLZC with $z_{0}$ and *b*.

The complexity of System (15) is analyzed by varying system parameter *b* from 0.525 to 0.725 with a step size 0.002 and setting $y_{0}=0.1+2k\pi$, $z_{0}=0$ or $y_{0}=0.1$, $z_{0}=2k\pi$, k∈[−25,25], k∈Z. It is shown in Figure 7 that the system has lower complexity when parameter *b* takes values larger than 0.55. Additionally, in these cases, there are no significant changes of complexity with the variation of $y_{0}$ and $z_{0}$. However, when parameter *b* takes values between 0.525 and 0.55, some lower complexity analysis results are observed. This illustrates that the complexity of the system is determined by the initial conditions. Moreover, compared with MMPE, MMLZC obtains better analysis results when the system is in the route from period to chaos.

**Figure 8:**
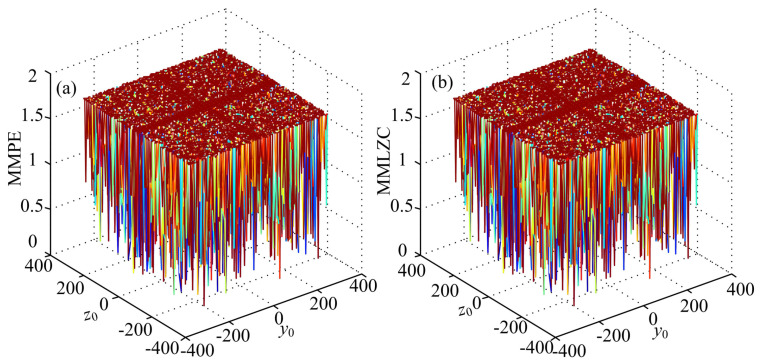
Complexity of System (15) with both $y_{0}$ and $z_{0}$ varying. (**a**) MMPE; (**b**) MMLZC.

**Figure 9:**
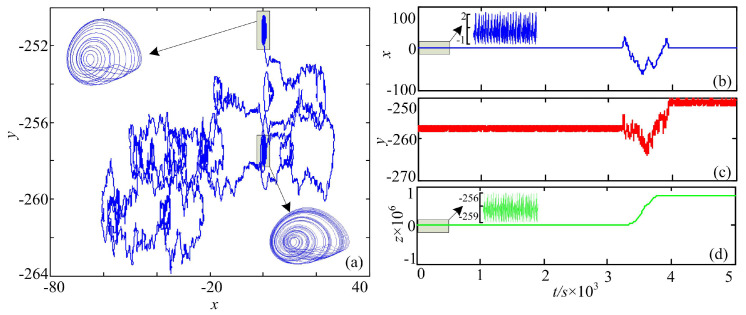
Dynamics of System (15) with $\left(x_{0},y_{0},z_{0}\right)=\left(0,0.1-82\pi ,-82\pi \right)$. (**a**) Phase diagram; (**b**) time series *x*; (**c**) time series *y*; (**d**) time series *z*.

Moreover, let $y_{0}=0.1+2k\pi$ and $z_{0}=2k\pi$, where k∈[−50,50] and k∈Z. The complexity analysis result of System (15) with different initial conditions is shown in Figure 8. It is shown that the system has high complexity in most cases. According to [12], when $y_{0}=0.1+2k\pi$ and $z_{0}=2k\pi$, chaotic attractors can be observed although in different positions. It is supposed to be a result that only high complexity could be found in this system. However, analysis results also indicate that the system could have a low complexity when $y_{0}$ and $z_{0}$ are varying. To explain this, phase portraits and time series of the system with $\left(x_{0},y_{0},z_{0}\right)=\left(0,0.1-82\pi ,-82\pi \right)$ are observed, and the results are shown in Figure 9. It is shown in Figure 9 that the system has chaotic attractors, but the fluctuation range of state variable *z* changes after a nonchaotic period t∈(3000s,4000s). In real practical applications, the nonchaotic period should be avoided, and complexity measure methods provide a method to fulfill this.

## 4. Discussion

### 4.1. Comparison with the Corresponding Original Systems

Actually, self-reproducing chaotic systems can be constructed based on the existing systems. System (13) is designed based on the following system [30]:

(16) $$\left\{\begin{array}{c} \dot{x}=\left|y\right|-1\\ \dot{y}=z\\ \dot{z}=x-by-az \end{array}\right.,$$

where a=0.6 and b=1. When solving this system, the initial condition is given by $\left(x_{0},y_{0},z_{0}\right)=\left(2,0,-1\right)$. The LE calculation results are (0.0363,0,−0.6363). System (14) is designed based on the following equation [31]:

(17) $$\left\{\begin{array}{c} \dot{x}=az+y^{2}-1\\ \dot{y}=byz\\ \dot{z}=-x-z \end{array}\right.,$$

where a=2.8 and b=4. System (17) has chaotic attractors with Lyapunov exponents (0.1149,0,−1.1149) under the initial condition when $\left(x_{0},y_{0},z_{0}\right)=\left(1,1,0\right)$.

The two-directional self-reproducing chaotic system is designed based on the following [32]:

(18) $$\left\{\begin{array}{c} \dot{x}=y\\ \dot{y}=z\\ \dot{z}=x^{2}-x-0.5\dot{y}-\dot{x} \end{array}\right.,$$

which gives offset boosting with an identical strange attractor with LEs (0.0938,0,−0.5938) under the initial condition $\left(x_{0},y_{0},z_{0}\right)=\left(13,-1,0\right)$.

The complexities of the original systems and the corresponding self-reproducing versions are given in Table 1. These systems are solved in the time interval [0,200] with a step size of 0.01. The complexity of System (13) is calculated with initial condition $\left(x_{0},y_{0},z_{0}\right)=\left(2+k\pi ,0,-1\;\left(k=-5,-4,\cdots ,5\right)\right)$, and its mean value of MMPE is 1.5579, while the mean value of MMLZC is 0.5376. Compared with the original System (16), System (13) with infinitely many attractors has a higher MMPE complexity, while a smaller maximum Lyapunov exponent (MLE). That is to say, the complexity of the modified self-reproducing system is almost the same as System (16). The initial conditions of System (14) are given in Figure 4. Table 1 shows that the modified system has higher complexity than its original system, which is given by Equation (17). Moreover, we compared the complexity of a two-directional self-reproducing chaotic system with its original system. It has higher MMLZC, but lower MLEs. In conclusion, our complexity analysis results illustrate that the new systems have relatively higher complexity. The effectiveness of the proposed methods and the potential application values in the fields including information encryption and secure communication of the proposed systems are shown.

### 4.2. Comparison of MMPE and MMLZC

**Figure 10:**
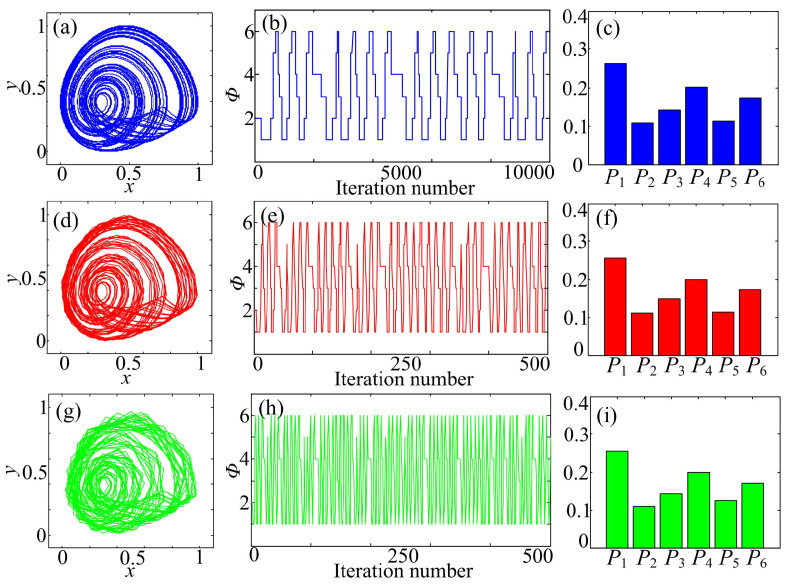
States of System (15) under different scale factors. (**a**) Phase diagram under τ=1; (**b**) quantification pattern series under τ=1; (**c**) probability distribution under τ=1; (**d**) phase diagram under τ=50; (**e**) quantification pattern series under τ=50; (**f**) probability distribution under τ=50; (**g**) phase diagram under τ=100; (**h**) quantification pattern series under τ=100; (**i**) probability distribution under τ=100.

It is shown in Figure 2 and Figure 4 that measuring values of MMPE does not change with the scale factor, while MMLZC increases with the scale factor. Generally, the multiscale complexity of a continues chaotic system increases with the scale factor [18,23,24,25]. However, when multivariate states are considered, the situation is different. Firstly, we take System (15) as an example to show the effectiveness of the scale factor on multivariate measuring algorithms. The parameters of System (15) are a=1.05 and b=0.525. The chaotic attractor of System (15) is shown in Figure 10. Since it is plotted from the system directly, it shows the scale factor τ=1. Moreover, we also plot the phase diagrams, quantification pattern series and probability distribution when the scale factor is revised to be τ=50 and 100. It is shown in Figure 10 that although phase diagrams under a larger, different scale factor become rough, their shapes are similar. This means that the relationship between different variables is not changed.

Let function φ(·) represent the comparison of the processing of Equation (3), then the pattern series for one time series and multiple time series can be obtained by:

(19) $$S_{One}^{\tau }\left(i\right)=\varphi \left(x_{i}^{\tau },x_{i+1}^{\tau },x_{i+2}^{\tau }\right),$$

and:

(20) $$S_{Mul}^{\tau }\left(i\right)=\varphi \left(x_{i}^{\tau },y_{i}^{\tau },z_{i}^{\tau }\right),$$

respectively, where $x^{\tau }$, $y^{\tau }$ and $z^{\tau }$ are the coarse-grained series under scale factor τ and i=1,2,⋯. Obviously, these two processes are different. As for MMPE, it is calculated based on the probability distribution of different patterns. Because the relationship is not changed, the probability distribution (statistical result) will keep the same, which is verified in Figure 10. Therefore, the values of MMPE do not change with the scale factor. However, MMLZC calculates complexity based on the new reproduction in the pattern series. When the scale factor is larger, it is easier to find the insertion, and the complexity measuring result is larger. This gives an explanation, at least to a significant degree, why values of MMPE do not change with the increase of the scale factor.

Currently, multiscale processing is a hot topic, and it is widely used in many research works. Scientists found that it indeed makes the complexity measuring results better. However, when it comes to multiple time series, the situation is different according to our analysis. One should carefully introduce this multiscale process for complexity analysis of a multivariate system, since it sees that it is not necessary for entropy-based algorithms.

However, it should be pointed out that the two proposed complexity measures are reliable and effective for the complexity analysis of chaotic attractors in the following aspects. In the first place, the pattern series is obtained based on the time series from different dimensions of the system. It can reflect the nature of the complexity of attractors directly. Secondly, MMPE is designed by employing the concept of permutation entropy and Shannon entropy, while MMLZC is proposed based on the Lempel–Ziv complexity. As we all know, the PE algorithm and Lempel–Ziv algorithm are mature algorithms and have been widely used by many researchers in a vast quantity of literature. Moreover, it is shown in the literature and in the above analysis results that the multi-scale process could make the complexity measuring results better. Thus, the two proposed complexity measuring algorithms have potential application value in real applications.

## 5. Conclusions

In this paper, MMPE and MMLZC are employed to analyze the complexity of multivariate systems, and the MMPE vs. MMLZC map is introduced to demonstrate the complexity. How the complexity of the self-reproducing systems is determined by the initial condition is investigated. Moreover, we found that the multiscale coarse graining process does not affect the final result of MMPE, but a good MMLZC measuring result can be obtained by choosing a proper large-scale factor.

Since the self-reproducing chaotic systems can generate strange attractors in different positions under various initial conditions, multivariate complexities including MMPE and MMLZC of these systems with different initial conditions are analyzed. It is shown that the complexity of self-reproducing systems depends on the initial conditions. Especially, transition stages can be found in the two-directional self-reproducing chaotic systems since in this case the system has relatively lower complexity analysis results. Moreover, compared with their corresponding original systems, the newly-developed multistable systems have the same level or a higher level of complexity. The theoretical and practical significance of the multistable systems and complexity measuring algorithms is shown. 

## Figures and Tables

**Table 1:** Complexity comparison in different systems.

(Original, New)	${LE}_{max}$	MMPE	MMLZC
(Sys(16), Sys(13))	(0.0363, 0.0285)	(1.3805, 1.5579)	(0.5402, 0.5376)
(Sys(17), Sys(14))	(0.1149, 0.1101)	(1.1610, 1.2657)	(0.5402, 0.5907)
(Sys(18), Sys(15))	(0.0938, 0.0890)	(1.7435, 1.7414)	(0.6483, 0.6976)

$LE_{max}$: maximum Lyapunov exponent; MMPE: multiscale multivariate permutation entropy; MMLZC: multiscale multivariate Lempel–Ziv complexity; Sys: System.
